# Divergent dynamics of nurse-patient trust during COVID-19 in China: the mediating effects of medical expectations and communication skills

**DOI:** 10.1186/s12912-025-03988-9

**Published:** 2025-10-31

**Authors:** Jingze Wang, Yaxuan Bi, Yanjiao Wang, Wenjing Yan, Yahui Yu, Pei Wang

**Affiliations:** 1 Faculty of Education, University of Macau, Macau, China; 2 Zhejiang Provincial Clinical Research Center for Mental Health, The Affiliated Kangning hospital of Wenzhou Medical University, Wenzhou, Zhejiang Province 325035 China; 3 School of Psychology, Inner Mongolia Normal University, Hohhot, China; 4 Department of Pediatric Respiratory Medicine, The Second Affiliated Hospital of Wenzhou Medical University, Wenzhou, 325035 China; 5 College of Medical Humanities and Management, Wenzhou Medical University, North Central Road, Chashan Higher Education Park, Ouhai District, Wenzhou, Zhejiang Province 325035 China; 6 Key Research Center of Philosophy and Social Sciences of Zhejiang Province, Institute of Medical Humanities, Wenzhou Medical University, Wenzhou, 325035 China; 7 College of Education, Shanghai Normal University, Shanghai, 210034 China; 8 School of Mental Health, Wenzhou Medical University, Wenzhou, 325000 China

**Keywords:** COVID-19, Nurse-patient trust, Medical expectations, Communication skills

## Abstract

**Background:**

There have been changes in nurse-patient trust in China during the COVID-19 pandemic. However, it remains unclear how nurse-patient trust changed during the pandemic compared to the pre-pandemic period, or what factors potentially influenced these changes.

**Objective:**

This study aimed to explore the changes in nurse-patient trust in China due to the COVID-19 pandemic, as well as the moderating roles of medical expectations and communication skills during this period.

**Design:**

Using convenience sampling, surveys were conducted among nurses and patients aged 18 and above with Chinese - language proficiency in 38 cities across 11 provinces from November 18 to December 8, 2019 (before the pandemic) and from February 1 to April 8, 2020 (during the pandemic).

**Participants:**

Data were obtained from a survey of 1883 nurses and 1850 patients in China.Chinese nurses and patients over 18 years old who could read Chinese, and the principle was that they would participate voluntarily and obtain oral consent.

**Methods:**

From 18 November 2019 to 8 December 2019, and from 1 February 2020 to on April 8 2020,participants of 38 cities in China on medical expectations, nurses’ communication skills and nurse-patient trust by Chinese Nurse Trust Scale, Chinese version of the Wake Forest Trust Scale, self-designed medical expectations scale and the Chinese version of the SEGUE Frame-work Communication Scale.

**Results:**

During the COVID-19 pandemic, nurses’ trust in patients significantly increased (t = -20.69, *p* < .001), while patients’ trust in nurses decreased significantly (*t* = 12.64, *p* < .001) compared to the pre-pandemic period.In addition to the influence of patients’ expectations on the nurse-patient trust, nurses’ expectations and communication skills and patients toward nurses had a significant impact on the change of the nurse patient trust.Before the pandemic, patients’ expectations were positively correlated with nurse - patient trust (β = 0.18, 95% CI [0.13, 0.24], *p* < .001), and nurses’ communication skills had a significant impact on patients’ trust (β = 0.41, 95% CI [0.36, 0.47], *p* < .001). During the pandemic, nurses’ expectations were negatively correlated with nurse-patient trust (β = -0.10, 95% CI [-0.19, -0.02], *p* = .02), while for patients, it was positively correlated (β = 0.16, 95% CI [0.11, 0.21], *p* < .001). Nurses’ communication skills still played an important mediating role in patients’ trust, but the mediating role in nurses’ own trust changed from non-significant to significantly negative (indirect effect = -0.08, SE = 0.03, 95% CI [-0.13, -0.02]).

**Conclusions:**

COVID-19 appears to have contributed to a ‘Divergent Dynamics’ in nurse-patient trust (defined as nurses and patients experiencing opposing trends in their perception of trust changes).This phenomenon seems to have influenced established patterns in the nurse-patient trust relationship, while also affecting the roles of medical expectations and communication skills. Expectations and communication skills may have a moderating effect on nurse-patient trust. This underscores the context-dependent necessity for emergency communication adaptations, providing psychological support for medical staff, and implementing targeted interventions during major public health events.

**Clinical trial number:**

Not applicable.

## Introduction

Nurse-patient trust serves as the cornerstone of therapeutic relationships and is fundamental to effective nursing practice [1]. A trusting relationship between nurses and patients not only enhances patient satisfaction and treatment compliance but also significantly improves clinical outcomes [2]. Furthermore, this trust acts as a powerful motivational mechanism for healthcare providers - nurses who experience patient trust demonstrate higher levels of engagement and deliver superior quality care compared to their counterparts who lack such trust relationships [3]. Therefore, establishing trusting nurse-patient relationships is essential.

However, nurse-patient trust within China’s healthcare system has deteriorated concerningly over the past decade. Frequent conflicts between healthcare providers and patients have precipitated a deepening crisis of trust, resulting in mutual distrust between Chinese nurses [4]and patients [5, 6]. Yi et al. [7]conducted a meta-analysis spanning ten years of surveys among Chinese medical staff, revealing consistently low levels of trust in patients among nurses. This defensive stance frequently triggers patient dissatisfaction and other negative emotional responses, potentially escalating into conflicts that further undermine the establishment of trusting relationships. These findings align with numerous patient-centered studies on nurse-patient trust dynamics [8].

A notable shift in this dynamic occurred during China’s initial COVID-19 outbreak in Wuhan. Chinese nurses demonstrated remarkable courage and dedication, while patients showed unprecedented cooperation, contributing to epidemic containment within five months [9]. During this period, patients gained profound appreciation for nurses’ comprehensive care responsibilities, including therapeutic interventions, life care, basic care, and psychosocial support [10]. Surveys conducted during the pandemic revealed significantly elevated levels of trust from patients toward nurses [11], with similarly high levels of trust from nurses toward patients [3].

This elevated mutual trust during the epidemic can be attributed largely to the Chinese government’s decisive response, including city-wide lockdowns and the mobilization of healthcare workers across provinces to provide assistance [12]. These measures significantly influenced the medical expectations of both nurses and patients. Despite facing increased workloads and considerable challenges during the pandemic - including psychological distress such as anxiety [13], fear [14], and concerns about personal safety [15] - nurses maintained professional expectations of patient cooperation and mutual respect [16]. Similarly, patients not only expected standard nursing care, including service provision, information sharing, and privacy protection, but also anticipated enhanced resilience from nurses during the crisis [17]. These heightened mutual expectations fostered greater attention and consideration between parties, facilitating patient-centered therapeutic processes and outcomes [18], and ultimately strengthening nurse-patient trust [16].

In the context of COVID-19, where common clinical manifestations include pain, dry cough, and fatigue, patients’ pain perception is notably influenced by their expectations [19]. When patient experiences align with anticipated outcomes, an assimilation effect occurs [20], influencing variables such as patient prognosis [21]. A meta-analysis of expectation interventions in chronic disease and post-surgical patients demonstrated that medical expectations can effectively reduce pain perception, with verbal suggestions from caregivers proving particularly effective [18, 22]. conducted a randomized controlled trial with 128 patients, revealing that those with higher expectations reported better pain experiences and greater trust in nurses, highlighting the potential impact of positive nurse communication on trust-building.

**Fig. 1:**
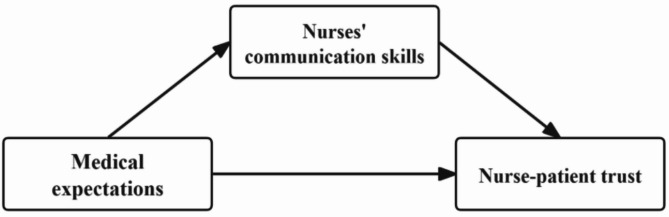
Theoretical framework

Clinical studies have consistently shown that nurse-patient communication encompasses multiple aspects such as the determination of nursing goals, disease-specific counseling [23], and shared decision-making [24]. For patients, effective communication with nurses can not only improve the quality of communication, but also enhance their understanding of prognosis, satisfaction, rehabilitation outcomes and trust. However, it remains unclear whether nurse-patient trust in China maintained its improvement during the period when the Chinese government implemented lockdown measures [10], and whether this improvement stemmed from mutual expectations and nurses’ communication skills. Based on our survey results of nurse-patient trust in China before and during the COVID-19 pandemic, we propose hypothesis 1: Compared with pre-pandemic levels, nurse-patient trust in China improved significantly during the COVID-19 pandemic. Furthermore, this study draws on Wang Pei et al.‘s theoretical framework, which suggests that healthcare professionals’ trust in patients consists of three core elements: preset expectations at the group level form the basis of trust, the quality of interpersonal communication in specific interactions plays an indirect role in trust, and stereotypes at the psychosocial level moderates the effects of the first two [25].we posit that medical expectations serve as a primary variable in building nurse-patient trust, directly affecting both trust outcomes and nurses’ communication skills. We propose that nurses’ communication skills mediate the relationship between medical expectations and nurse-patient trust. Based on this research framework (Fig. 1), we hypothesize that both before and during the epidemic, nurses’ and patients’ expectations had a significant positive impact on nurse-patient trust (H2a). Moreover, nurses’ and patients’ expectations significantly influence nurse-patient trust through nurses’ communication skills (H2b).

## Methods

This study employed a cross-sectional design to investigate changes in nurse-patient trust dynamics in China before and during the initial outbreak of COVID-19. The study specifically examined potential variations in trust levels, alongside the mediating effects of medical expectations and nurses’ communication competencies during this period.

### Participants and sampling

Using convenience sampling methodology, we conducted extensive surveys across China both before and during the first wave of COVID-19. The pre-pandemic survey was administered between November 18 and December 8, 2019. Following the World Health Organization’s declaration of COVID-19 as a public health emergency on January 30, 2020, the during-pandemic survey commenced on February 1, 2020, and concluded upon the lifting of Wuhan’s lockdown on April 8, 2020 [26].

Trained hospital personnel distributed questionnaires to nurses and patients within our affiliated healthcare facilities. Eligible participants included Chinese nurses and patients aged 18 years or older with Chinese language proficiency. Participation was voluntary, and oral consent was obtained from all participants.

The surveys encompassed 38 cities across 11 provinces, including major metropolitan areas (Beijing, Shanghai) and diverse regions (Heilongjiang, Liaoning, Anhui, Jiangsu, Zhejiang, Hunan, Yunnan, Hainan, and Xinjiang). Healthcare facilities ranging from primary to tertiary institutions were included, representing varying levels of hospital capacity and capability.

The final sample comprised 3,733 valid responses: 1,883 from nurses (1,098 pre-pandemic, 785 during-pandemic) and 1,850 from patients (983 pre-pandemic, 867 during-pandemic). The nurse cohort had a mean age of 31.14 ± 7.23 years, with 78.0% being female. The patient group averaged 40.05 ± 14.76 years, with 54.0% female representation. Notably, 72% of nurses and 81% of patients were affiliated with tertiary hospitals, while 95% of nurses and 53% of patients had completed tertiary education or higher. The study received approval from the Ethics Committee of Shanghai Normal University.

### Measures

Nurse-patient Trust Assessment. Nurses’ trust in patients was measured using the Chinese Nurse Trust Scale (C-NTS), while patients’ trust in nurses was evaluated through the Chinese version of the Wake Forest Trust Scale (C-WFPTS) [27]. The C-NTS comprises 12 items assessing nurses’ perceptions of patient behavior and reliability, including statements such as “The patient will follow the treatment plan you suggest” and “The patient will not make unreasonable demands.” The C-WFPTS contains 10 items evaluating patients’ trust in healthcare professionals, featuring statements like “To ensure my health, the medical staff will help me as much as possible” and “I found my doctor very approachable.” Both scales employ a 5-point Likert format (1="strongly disagree” to 5="strongly agree”), with total scores ranging from 10 to 50. Higher scores indicate greater trust levels. The scales demonstrated robust internal consistency with Cronbach’s Alpha coefficients of 0.92 and 0.80 respectively.

Medical Expectations Measurement. A self-designed medical expectations scale was developed with distinct sections for medical staff and patients.The scale was validated for good construct validity in another study [25].The nurses’ section contained three items assessing their expectations of patient behavior, exemplified by statements such as “I want the patient to follow doctors’ instructions.” The patients’ section included four items evaluating their expectations of nursing care, such as “I expect nurses to treat me kindly.” Both utilized 5-point Likert scales, with higher scores indicating elevated expectation levels. The scales showed strong reliability with Cronbach’s Alpha coefficients of 0.89 for nurses and 0.91 for patients.

Communication Skills Assessment. The Chinese version of the SEGUE Framework Communication Scale was administered to both nurses and patients [28]to evaluate nurses’ communication competencies. The scale consists of 25 items, with content tailored to each respondent group. For instance, the nurse item “treat patients politely when visiting” corresponds to the patient item “the nurse will greet me politely when visiting.” The 5-point Likert scale ranged from “never” to “all the time,” with higher scores indicating superior communication skills. The scales demonstrated excellent internal consistency with Cronbach’s Alpha coefficients of 0.96 for nurses and 0.97 for patients.

### Ethical consideration

This study received approval from the University Ethics Committee. All participants were informed of the voluntary nature of their participation and their right to withdraw at any time without explanation. Oral consent was obtained from each participant prior to data collection. The study maintained strict adherence to research ethics principles, ensuring participant anonymity and confidentiality throughout the process.

### Data analysis

All participants completed the questionnaire through online answering. After manually recording the collected data, the research team excluded questionnaires with response times outside three standard deviations from the mean response time or the answering time over ± 3 standard deviations (SD) of the mean or the response with cognitive bias.Data analysis was conducted using Excel 2019 and Stata16.0, following four sequential analytical phases that aligned with our research objectives.

First, we performed descriptive statistical analysis to examine the distribution characteristics and correlations of all variables. This included analyzing the normality of distribution through skewness and kurtosis, and calculating correlation coefficients between variables for both the pre-pandemic and during-pandemic periods.

Second, we conducted independent sample T-tests to investigate changes in three key variables (nurse-patient trust, medical expectations, and nurses’ communication skills) before and during the epidemic. This analysis was performed separately for nurses and patients to identify group-specific changes.

Third, we examined the relationships among the three variables and their temporal changes through multiple regression analysis. Prior to analysis, all variables were standardized. The analysis proceeded in three steps while controlling for demographic variables: (1) examining the influence of medical expectations on nurse-patient trust, (2) investigating the impact of nurses’ communication skills on nurse-patient trust, and (3) analyzing the effect of medical expectations on nurses’ communication skills. The Chow test was then employed to determine whether these relationships significantly differed between the pre-pandemic and during-pandemic periods.

Finally, we conducted mediation analysis to understand how nurses’ communication skills mediated the relationship between expectations and nurse-patient trust. This analysis was performed separately for the pre-pandemic and during-pandemic periods, using bootstrap methodology with 5000 resamples. The mediation effect was considered significant if the 95% confidence interval excluded zero. Effect sizes were calculated to quantify the relative importance of the mediating pathway.

## Results

### Descriptive statistics

The descriptive statistical analysis and correlations between core variables for both nurses and patients before and during the epidemic are presented in Table 1. All variables demonstrated normal distribution patterns. Significant correlations were observed between most variables, with one notable exception - the lack of correlation between nurses’ medical treatment expectations and their self-evaluated communication ability before the epidemic. This pattern of correlations suggested potential mediating relationships among these variables.

**Table 1:** Descriptive statistics and correlations of indicators before and during COVID-19

Participants	Variables	*M*	*SD*	Skewness	Kurtosis	1	2
Nurses	Expectations-B	13.40	1.51	-0.10	-1.36		
	Communication-B	110.64	12.30	-0.53	-0.51	0.32^**^	
	Nurse-patient trust-B	32.21	9.18	0.56	0.24	0.05	0.05
	Expectations-D	11.90	1.70	-0.22	1.27		
	Communication-D	94.78	12.50	0.42	0.39	0.62^**^	
	Nurse-patient trust-D	40.05	7.25	-0.84	0.01	-0.18^**^	-0.19^**^
Patients	Expectations-B	17.50	2.04	0.03	-1.07		
	Communication-B	102.63	15.54	-0.54	0.71	0.46^**^	
	Nurse-patient trust-B	39.10	5.29	0.35	0.17	0.37^**^	0.50^**^
	Expectations-D	16.76	2.33	-0.66	1.89		
	Communication-D	92.18	15.58	-0.60	0.93	0.23^**^	
	Nurse-patient trust-D	36.19	4.61	0.13	0.56	0.29^**^	0.59^**^

### Temporal changes in key variables during the COVID-19 pandemic

**Fig. 2:**
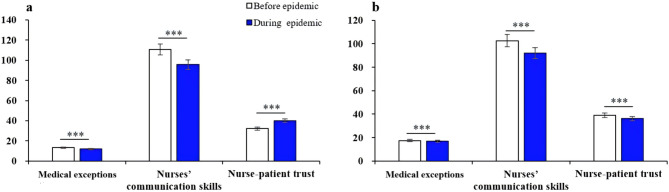
Changes in nurse-patient trust, expectations and nurses’ communication skills before and during the epidemic. Note: **a** and **b** are nurses and patients result, respectively

Comparative analysis revealed distinct patterns of change in nurse-patient trust, medical expectations, and communication skills before and during the epidemic. From the nurses’ perspective (Fig. 2a), their trust in patients increased significantly during the epidemic compared to pre-epidemic levels (*t* = -20.69, *p* < .001). However, they reported lower self-evaluated communication skills (*t* = 27.34, *p* < .001) and reduced expectations (*t* = 19.74, *p* < .001) during this period.

In contrast, patients’ evaluations (Fig. 2b) showed a consistent decline across all three variables during the pandemic. Specifically, nurse-patient trust (*t* = 12.64, *p* < .001), perceived nurses’ communication skills (*t* = 14.41, *p* < .001), and medical expectations (*t* = 7.22, *p* < .001) were all significantly lower compared to pre-pandemic levels.

### Relationship dynamics between core variables pre-epidemic

**Fig. 3:**
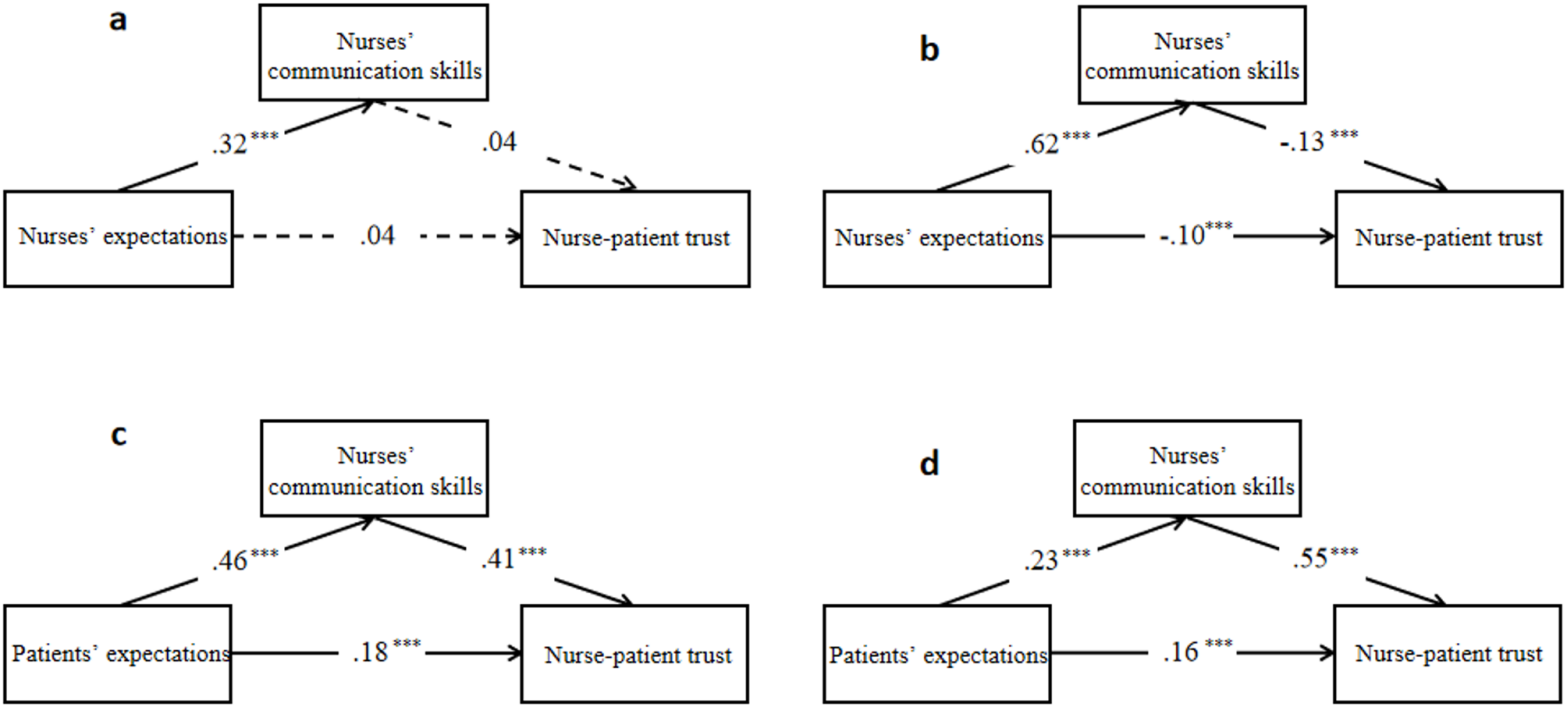
The relationship between nurses and patients before and during the epidemic. Note: **a** and **b** are standardized regression coefficients between the three variables before and during the epidemic for nurses, and **c** and **d** are standardized regression coefficients between the three variables before and during the epidemic for patients, respectively. A solid line means significant, and a dotted line means insignificant

Further analysis examined the interrelationships between nurse-patient trust, medical expectations, and nurses’ communication abilities before the COVID-19 outbreak through a mediation model (Fig. 3). From the nurses’ perspective, their expectations did not significantly influence nurse-patient trust (β = 0.04, 95% CI [-0.02, 0.10], *p* > .05). In contrast, patients perceived a significant positive relationship between these variables (β = 0.18, 95% CI [0.13, 0.24], *p* < .001). Regarding communication skills’ impact, nurses did not perceive their communication abilities as significantly affecting nurse-patient trust (*p* > .05). However, patients reported a substantial positive influence (β = 0.41, 95% CI [0.36, 0.47], *p* < .001). Both groups acknowledged that expectations significantly influenced nurses’ communication skills, with positive correlations reported by both nurses (β = 0.32, 95% CI [0.27, 0.38], *p* < .001) and patients (β = 0.46, 95% CI [0.41, 0.51], *p* < .001).

### Evolving relationships during the pandemic

During the COVID-19 pandemic, both nurses (β = -0.10, 95% CI [-0.19, -0.02], *p* = .02) and patients (β = 0.16, 95% CI [0.11, 0.21], *p* < .001) reported significant relationships between expectations and nurse-patient trust, though in opposite directions (Fig. 3). The impact of communication skills on nurse-patient trust showed similar divergence: nurses reported a negative association (β = -0.13, 95% CI [-0.21, -0.04], *p* < .01), while patients maintained a strong positive relationship (β = 0.55, 95% CI [0.50, 0.60], *p* < .001). The influence of expectations on communication skills remained consistently positive for both nurses (β = 0.62, 95% CI [0.58, 0.66], *p* < .001) and patients (β = 0.23, 95% CI [0.17, 0.29], *p* < .001), though with different magnitudes.

### Comparative analysis of pre- and during-pandemic relationships

The Chow test was employed to examine the significance of temporal changes in relationships between expectations, communication skills, and nurse-patient trust (Table 2). A notable finding was that from the patients’ perspective, the influence of nurses’ communication skills on nurse-patient trust remained stable across both periods. However, all other relationship variables demonstrated significant changes for both nurses and patients, indicating substantial shifts in the dynamics of nurse-patient relationships during the pandemic.

**Table 2:** Results of chow’s test

Participants	Relation between variables	Coefficient	*p*	*95%CI*
Nurses	Expectation→Nurse-patient trust	-1.11	< 0.001	[-1.58, -0.63]
	Nurses’ communication skills→Nurse-patient trust	-0.15	< 0.001	[-0.21, -0.09]
	Expectation→Nurses’ communication skills	1.94	< 0.001	[1.31, 2.56]
Patients	Expectation→Nurse-patient trust	-0.40	< 0.001	[-0.60, -0.20]
	Nurses’ communication skills→Nurse-patient trust	0.01	0.69	[-0.02, 0.03]
	Expectation→Nurses’ communication skills	-1.96	< 0.001	[-2.57, -1.35]

### Mediating effects of communication skills

The final analysis investigated the mediating role of nurses’ communication skills in the relationship between expectations and nurse-patient trust (Table 3). From the nurses’ perspective, no significant mediating effect was observed before the epidemic (indirect effect = 0.01, *SE* = 0.01, 95% *CI* [-0.01, 0.03]). However, during the epidemic, a significant negative mediating effect emerged (indirect effect = -0.08, *SE* = 0.03, 95% *CI* [-0.13, -0.02]) with a mediating effect size of 0.45. From the patients’ perspective, nurses’ communication skills consistently demonstrated significant mediating effects both before (indirect effect = 0.19, *SE* = 0.02, 95% *CI* [0.16, 0.22]) and during the epidemic (indirect effect = 0.13, *SE* = 0.02, 95% *CI* [0.09, 0.16]). The mediating effect sizes were substantial at 0.51 and 0.44 respectively, indicating that communication skills significantly mediated the translation of patients’ expectations into trust outcomes throughout both periods. These findings highlight the complex and evolving nature of nurse-patient relationships during the COVID-19 pandemic, with particular emphasis on the divergent perspectives of nurses and patients, and the crucial mediating role of communication skills in maintaining trust relationships.

**Table 3:** The mediating effect of nurses’ communication skills on expectations and nurse-patient trust before and during the epidemic

Participants	Results	Indirect effect Coefficient (SE)	95%CI	Effect size
Nurses	Before the epidemic	0.01 (0.01)	[-0.01, 0.03]	-
	During the epidemic	-0.08 (0.03)	[-0.13, -0.02]	0.44
Patients	Before the epidemic	0.19 (0.02)	[0.16, 0.22]	0.51
	During the epidemic	0.13 (0.02)	[0.09, 0.16]	0.45

## Discussion

This large-scale survey across 38 Chinese cities revealed key findings about nurse-patient trust during the pandemic. First, we observed a notable divergence: nurses reported increased trust in patients, while patients paradoxically showed decreased trust in nurses. Second, both groups had significantly lower medical expectations and communication skills during versus before the pandemic. Third, mediation analysis showed nurses’ communication skills consistently mediated the expectation-trust relationship for patients in both periods, whereas this mediating effect varied temporally for nurses. Crucially, the pandemic disrupted traditional trust-building pathways, particularly affecting how nurses’ communication skills influenced patient trust.

Furthermore, the sustained post-epidemic rise in nurses’ trust toward patients reconfigures social exchange theory in crises. Traditional theory holds that trust stems from predictable reciprocity [29], suggesting physical isolation should reduce interactions and decay trust. However, nurses observed that patients who cooperatively endured visitation restrictions and illness-related loneliness—despite receiving no direct rewards—reduced nursing workloads by maintaining healthcare order. This led nurses to view patients as allies against shared threats [30], not as resource takers in transactional relationships. This highlights how crisis-driven altruistic sacrifice for collective goals can transcend immediate reciprocity to build trust.

### Divergent patterns in nurse-patient trust

Our results demonstrate a notable divergence in trust perceptions between nurses and patients during the COVID-19 pandemic. While nurses reported increased trust in patients during the pandemic, patients paradoxically showed decreased trust in nursing staff. Festinger’s theory of cognitive dissonance states that individuals reduce psychological discomfort by adjusting their cognitions. During the epidemic, nurses tended to interpret patient anxiety or dissatisfaction as an understanding of the stress of the epidemic rather than distrust directed at the individual nurse, and this cognitive adjustment aligned them with their own hard-working behaviors and alleviated the dysfunction of putting in hard work without feeling trust [31], while patients experienced a decrease in the quality of care or emotional connectivity due to direct experience, and patients did not have the cognitive dissonance of needing to rationalize the nurse’s behavior, which directly contributes to a decrease in patient-reported trust.The enhanced trust from nurses aligns with previous research findings [3, 32]. However, our patient trust results contrast with [33]findings, possibly due to our more comprehensive geographical sampling approach. While Zhou et al.‘s study was limited to a specific region in mid-March 2020, our research encompassed 38 cities across 11 provinces, providing a more representative picture of patient perspectives throughout China. The increased trust from nurses likely reflects their recognition of patients’ active cooperation during the pandemic. However, the decline in patient trust may be attributed to the psychological impact of the pandemic on nurses, including fear and other negative emotions [10], which potentially affected nursing behavior and care quality [4], subsequently influencing patients’ perceptions of nurse-patient relationships.

### Decline in medical expectations and communication skills

A particularly noteworthy finding was the significant decline in both nurses’ and patients’ expectations and nurses’ communication skills during the pandemic compared to pre-pandemic levels. This decline can be attributed to several interrelated factors. First, the increased complexity of care responsibilities during the pandemic substantially reduced the time available for nurse-patient communication, a critical factor in maintaining effective healthcare relationships [34]. Additionally, the necessary use of protective equipment created physical barriers to communication, significantly impacting its effectiveness [35]. Furthermore, research has shown that the pandemic induced negative emotional states among nurses, including fear and anxiety, which affected their cognitive functioning and behavioral performance [36, 37].

Our findings suggest significant spillover and crossover effects in the healthcare communication ecosystem during this period. The impact on nurses’ communication abilities appears to have created a spillover effect, resulting in both nurses and patients reporting lower evaluations of nursing communication skills. Moreover, the observed decrease in medical expectations demonstrated a crossover effect between nurses and patients, aligning with Luhmann’s theory of reciprocal expectations in healthcare relationships. In Luhmann’s theory, medical expectation is the core medium that connects the patient-physician interaction, the healthcare organization and the overall healthcare system, and the decline in medical expectation observed during the epidemic is a reflection of the weakened function of this medium [38].When healthcare workers’ ability to communicate is severely compromised by work overload and protective limitations, such as reduced bedside communication and simplified explanations of conditions, bi-directional expectations at the interactive level are not effectively communicated and confirmed, patients’ care needs are not responded to, and the professional efforts of healthcare workers are not understood. The patient’s expectations are lowered due to disconnectedness, and communication difficulties are exacerbated by the lack of feedback, creating a vicious cycle. Luhmann’s perspective reveals that a decrease in expectations is not only an outcome, but also an adaptive signal from the system after the failure of the connecting medium.These effects highlight how the challenges faced by healthcare providers during the pandemic created a cascading impact on the entire healthcare communication system, affecting both the providers’ ability to communicate effectively and patients’ expectations and perceptions of care quality.

### Relationship dynamics between expectations and trust

The relationship between expectations and nurse-patient trust revealed interesting patterns. From the patients’ perspective, their expectations consistently showed a positive correlation with nurse-patient trust both before and during the pandemic, validating hypothesis 2a and supporting existing theories on medical care expectation differentials [39]. However, nurses’ experiences during the pandemic presented a contrasting pattern, consistent with [10]qualitative study of nurses in Wuhan. While nurses maintained high expectations for patient care responsibility, the combination of increased workload, infection risks, and reduced staffing during the public health emergency [40] created significant challenges. Our quantitative findings demonstrate that despite nurses’ continued commitment to patient care and recovery during the pandemic, the psychological burden and stress inherent in the crisis impeded their ability to build and maintain trusting relationships with patients.

### The mediating role of communication skills

The role of communication skills in trust-building also showed distinct patterns between groups. Patients consistently identified nurses’ communication skills as a significant factor affecting trust relationships both before and during the pandemic, supporting communication accommodation theory [41] and aligning with previous research on healthcare provider communication [24]. However, nurses’ perspectives on the impact of their communication skills evolved during the pandemic. While they perceived no significant relationship between communication skills and patient trust before COVID-19, they reported a negative correlation during the pandemic. This shift likely reflects the practical challenges of maintaining effective communication while managing increased workloads and navigating the barriers posed by protective equipment, which affected both verbal clarity and emotional expression [35, 42].

The mediating effect of nurses’ communication skills on the relationship between expectations and trust presents another layer of complexity in our findings. For patients, communication skills consistently mediated the relationship between expectations and trust, both before and during the pandemic, supporting hypothesis 2b. This finding reinforces the crucial role of effective nurse communication in translating patient expectations into trust-building outcomes [43]. However, from the nurses’ perspective, the mediating effect of communication skills showed temporal variation. While present before the pandemic, this mediating effect disappeared during the crisis, suggesting that the extraordinary circumstances of COVID-19 disrupted the traditional pathways through which nurses build trust with their patients.In the high-pressure environment of the new crown epidemic, nurses often mistake efficient delivery of medical information for improved communication skills, yet the core needs of patients are really emotional support and empathic response. At the same time, physical barriers such as masks and face screens severely diminish critical nonverbal communication such as smiles and glances, forcing nurses to compensate more frequently with verbal communication [1]. Nurses’ language tends to be streamlined when they are tired, which can easily be interpreted by patients as apathetic attitudes. Nurses’ efforts to overcome communication barriers instead lead to a self-perceived increase in communication skills, a perceived discrepancy that ultimately widens the gulf of understanding between doctors and patients.

### Implications for healthcare policy and practice

Norwegian data show a systematic strengthening of public trust in the healthcare system [44], whereas an Indian study reported that more than 80% of participants maintained trust in their doctors, but revealed that more than 60% of the population faced barriers to both access and communication, and that difficulties in communication led to a significant negative trust effect [45]. In stark contrast, the Chinese context shows a unique polarization of trust dimensions, with collectivist values and the “white angel” narrative promoting trust in nurses, while medical overload and segregation resulting from the “dynamic zero” policy continue to erode patient trust. This highlights the urgent need for developing countries to build adaptive communication technologies and resource transparency mechanisms to effectively transfer professional competence to public trust.

These findings have important implications for healthcare policy and practice. First, they highlight the need for healthcare systems to develop more resilient communication strategies that can withstand the challenges posed by public health emergencies. This might include implementing enhanced communication protocols and utilizing innovative technologies to facilitate nurse-patient interaction when physical barriers are necessary. Second, our results emphasize the importance of providing comprehensive psychological support for healthcare workers during crises, as their emotional well-being directly impacts their ability to maintain effective communication and build trust with patients. Third, the divergent trends in nurse and patient trust suggest the need for targeted interventions that address the specific concerns and needs of each group during public health emergencies.

### Limitations and future directions

This study has several limitations. First, the future study should expand the geographical coverage to better represent healthcare environments across diverse socioeconomic strata in China—particularly rural and resource-limited settings whose systemic challenges differ—so as to further test the present findings and enhance external validity. Second, the cross-sectional design precludes definitive causal inferences regarding the relationships between expectations, communication, and trust, as it cannot establish temporal order. Future longitudinal work is therefore essential to trace the developmental trajectory of trust. Furthermore, qualitative exploration is needed to uncover the contextualized mechanisms and nuanced interpersonal processes through which specific communication strategies and individualized expectations interact to shape trust in actual clinical practice.

## Conclusion

Nurses and patients exhibited some divergent perspectives on nurse-patient trust before and during the COVID-19 pandemic. Moreover, during the COVID-19 pandemic, both nurses and patients had decreased expectations for each other’s medical treatment, which showed a cross-effect, and the decreased communication ability of nurses showed a spillover effect. In addition, there were similarities and differences between nurses and patients on the effects of medical expectations and nurses’ communication skills on nurse-patient trust before and during the epidemic. No matter before or during the epidemic, medical expectation is a strong and positive influence factor on nurses’ communication skills for both nurses and patients. However, for patients, nurses’ communication skills still play a more influential role in nurse-patient trust than their medical expectations. Consequently, these findings suggest potential benefits of targeted communication training for nurses during crises. In the process of nursing, clear expectations of patients, and appropriate to patients to show their expectations of patients, is also conducive to the promotion of nurse-patient trust.

## Data Availability

The raw data generated in this study involving patient privacy is not available to the public. Non-sensitive data that have been de-identified may be obtained from the corresponding author upon reasonable request, subject to ethical norms. Data requesters are required to sign a Data Use Agreement and commit to use the data only to validate the results of this study.

## References

[1] Aguirre S, Jogerst KM, Ginsberg Z, Voleti S, Bhullar P, Spegman J, et al. COVID-19 impact on the Doctor-Patient relationship: patient perspectives on emergency physician empathy and communication. Bull Emerg Trauma. 2021;9:125–32. 10.30476/BEAT.2021.89058.1216. 34307702 10.30476/BEAT.2021.89058.1216 PMC8286650

[2] Strandås M, Bondas T. The nurse-patient relationship as a story of health enhancement in community care: A meta-ethnography. J Adv Nurs. 2018;74:11–22. 10.1111/jan.13389. 28702952 10.1111/jan.13389

[3] Shen L, Fei X, Zhou Y, Wang J, Zhu Y, Zhuang Y. The effect of felt trust from patients among nurses on attitudes towards nursing service delivery. J Adv Nurs. 2022;78:404–13. 10.1111/jan.14973. 34363632 10.1111/jan.14973

[4] Zhang L, Chai L, Zhao Y, Wang L, Sun W, Lu L, et al. Burnout in nurses during the COVID-19 pandemic in china: new challenges for public health. Biosci Trends. 2021;15:129–31. 10.5582/bst.2021.01099. 33776019 10.5582/bst.2021.01099

[5] Feng J, Lei Z, Li X, Qu G, Sun Y, Zheng Y, et al. Acceptance of family Doctors among residents in china: a cross-sectional study. Front Med (Lausanne). 2024;11:1435940. 10.3389/fmed.2024.1435940. 39301487 10.3389/fmed.2024.1435940 PMC11410576

[6] Ruan Y, Zhuang C, Chen W, Xie J, Zhao Y, Zhang L, et al. Limited knowledge and distrust are important social factors of out-patient’ s inappropriate diagnosed seeking behaviour: a qualitative research in Shanghai. Int J Health Plann Manage. 2021;36:847–65. 10.1002/hpm.3134. 33615549 10.1002/hpm.3134

[7] Yi X, Sicheng X, Lihui Z, Jianhui X, Zhenhui S, Xiang D, et al. Changes in empathy of nurses from 2009 to 2018: A cross-temporal meta-analysis. Nurs Ethics. 2021;28:776–90. 10.1177/0969733020968163. 33283617 10.1177/0969733020968163

[8] Liu PL, Jiang S. Patient-Centered communication mediates the relationship between health information acquisition and patient trust in physicians: A Five-Year comparison in China. Health Commun. 2021;36:207–16. 10.1080/10410236.2019.1673948. 31617412 10.1080/10410236.2019.1673948

[9] Liu Y-E, Zhai Z-C, Han Y-H, Liu Y-L, Liu F-P, Hu D-Y. Experiences of front-line nurses combating coronavirus disease-2019 in china: A qualitative analysis. Public Health Nurs. 2020;37:757–63. 10.1111/phn.12768. 32677072 10.1111/phn.12768 PMC7405388

[10] Liu Q, Luo D, Haase JE, Guo Q, Wang XQ, Liu S, et al. The experiences of health-care providers during the COVID-19 crisis in china: a qualitative study. Lancet Glob Health. 2020;8:e790–8. 10.1016/S2214-109X(20)30204-7. 32573443 10.1016/S2214-109X(20)30204-7 PMC7190296

[11] Tang X, Lu J, Chen Z, Liu C, Jiang X, Ning M. Influencing factors of patients’ trust in nurses during the COVID-19 pandemic: A Mixed-Methods study. Disaster Med Public Health Prep. 2022;17:e302. 10.1017/dmp.2022.262. 36325834 10.1017/dmp.2022.262 PMC9947035

[12] Wong LP, Wu Q, Hao Y, Chen X, Chen Z, Alias H, et al. The role of institutional trust in preventive practices and treatment-seeking intention during the coronavirus disease 2019 outbreak among residents in Hubei, China. Int Health. 2022;14:161–9. 10.1093/inthealth/ihab023. 33945613 10.1093/inthealth/ihab023 PMC8135875

[13] Liu J, Zheng Z, Ge L, Huang Y, Yang Q, Chen Y, et al. Reliability and validity of the Mandarin version of the trust in nurses scale. J Nurs Manag. 2022;30:1366–75. 10.1111/jonm.13623. 35403295 10.1111/jonm.13623

[14] Broos HC, Llabre MM, Saab PG, Leite RO, Port JH, Timpano KR. The relationship between health worry, work distress, and affective symptoms during the COVID-19 pandemic: the mediating role of hopelessness and helplessness. Br J Clin Psychol. 2023;62:10–27. 10.1111/bjc.12391. 36125014 10.1111/bjc.12391 PMC9538047

[15] Balkhi F, Nasir A, Zehra A, Riaz R. Psychological and behavioral response to the coronavirus (COVID-19) pandemic. Cureus. 2020;12:e7923. 10.7759/cureus.7923. 32499970 10.7759/cureus.7923 PMC7265762

[16] Evgin D, Şener Taplak A. Being a nurse during a worldwide pandemic: A qualitative study exploring nurses’ perceived challenges and expectations during the COVID-19 pandemic in Turkey. Disaster Med Public Health Prep. 2022;17:e239. 10.1017/dmp.2022.171. 35912633 10.1017/dmp.2022.171

[17] Jiménez-Fernández R, Corral-Liria I, Trevissón-Redondo B, Lopez-Lopez D, Losa-Iglesias M, Becerro-de-Bengoa-Vallejo R. Burnout, resilience and psychological flexibility in frontline nurses during the acute phase of the COVID-19 pandemic (2020) in Madrid, Spain. J Nurs Manag. 2022;30:2549–56. 10.1111/jonm.13778. 36042534 10.1111/jonm.13778 PMC9539113

[18] Peerdeman KJ, van Laarhoven AIM, Keij SM, Vase L, Rovers MM, Peters ML, et al. Relieving patients’ pain with expectation interventions: a meta-analysis. Pain. 2016;157:1179–91. 10.1097/j.pain.0000000000000540. 26945235 10.1097/j.pain.0000000000000540

[19] Müßgens D, Burgard LC, Kleine-Borgmann J, Frettlöh J, Sorgatz H, Bingel U. Impact of the COVID-19 pandemic on patients with chronic pain in germany: associations with expectations and control beliefs. Eur J Pain. 2022;26:1343–54. 10.1002/ejp.1955. 35445510 10.1002/ejp.1955 PMC9087415

[20] Peerdeman KJ, Geers AL, Della Porta D, Veldhuijzen DS, Kirsch I. Underpredicting pain: an experimental investigation into the benefits and risks. Pain. 2021;162:2024–35. 10.1097/j.pain.0000000000002199. 33470747 10.1097/j.pain.0000000000002199

[21] Laferton JAC, Schiller S, Conrad D, Fischer D, Zimmermann-Viehoff F. Stress beliefs moderate the impact of COVID-19 related work stress on depressive, anxiety and distress symptoms in health care workers. Stress Health. 2024;40:e3410. 10.1002/smi.3410. 38642346 10.1002/smi.3410

[22] van Vliet LM, Godfried MB, van Deelen GW, Kaunang M, Kaptchuk TJ, van Dulmen S, et al. Placebo effects of nurses’ communication alongside standard medical care on pain and other outcomes: A randomized controlled trial in clinical tonsillectomy care. Psychother Psychosom. 2020;89:56–8. 10.1159/000503904. 31655817 10.1159/000503904

[23] Wittenberg E, Ferrell B, Goldsmith J, Buller H, Neiman T. Nurse communication about goals of care. J Adv Pract Oncol. 2016;7:146–54. 10.6004/jadpro.2016.7.2.2. 28090365 10.6004/jadpro.2016.7.2.2 PMC5226308

[24] Expósito-Jiménez A, Alcaide-Leyva JM, Jiménez-Mérida MDR, Martínez-Angulo P. Health communication and shared decision-making between nurses and older adults in community setting: an integrative review. J Clin Nurs. 2024;33:2922–35. 10.1111/jocn.17152. 38573001 10.1111/jocn.17152

[25] Wang Y, Wu Q, Wang Y, Wang P. The formation mechanism of trust in patient from healthcare professional’s perspective: a conditional process model. J Clin Psychol Med Settings. 2022;29:760–72. 10.1007/s10880-021-09834-9. 35048251 10.1007/s10880-021-09834-9

[26] Jiaxin C, Hui H, Feifei W, Mi Z, Ting Z, Shicheng Y, et al. Air quality characteristics in Wuhan (China) during the 2020 COVID-19 pandemic. Environ Res. 2021;195:110879. 10.1016/j.envres.2021.110879. 33607094 10.1016/j.envres.2021.110879 PMC8479542

[27] Wu Q, Jin Z, Wang P. The relationship between the physician-Patient relationship, physician Empathy, and patient trust. J Gen Intern Med. 2022;37:1388–93. 10.1007/s11606-021-07008-9. 34405348 10.1007/s11606-021-07008-9 PMC9086002

[28] Guo A, Wang P. The current state of doctors’ communication skills in Mainland China from the perspective of doctors’ Self-evaluation and patients’ evaluation: A Cross-Sectional study. Patient Educ Couns. 2021;104:1674–80. 10.1016/j.pec.2020.12.013. 33384190 10.1016/j.pec.2020.12.013

[29] Keysar B, Converse BA, Wang J, Epley N. Reciprocity is not give and take: asymmetric reciprocity to positive and negative acts. Psychol Sci. 2008;19:1280–6. 10.1111/j.1467-9280.2008.02223.x. 19121138 10.1111/j.1467-9280.2008.02223.x

[30] Romero-García M, Delgado-Hito P, Gálvez-Herrer M, Ángel-Sesmero JA, Velasco-Sanz TR, Benito-Aracil L, et al. Moral distress, emotional impact and coping in intensive care unit staff during the outbreak of COVID-19. Intensive Crit Care Nurs. 2022;70:103206. 10.1016/j.iccn.2022.103206. 35120794 10.1016/j.iccn.2022.103206 PMC8776502

[31] Lu Q, Tan Y, Peng X, Tao L. Cognitive dissonance in the process of stress coping: front-line healthcare professionals’ information anxiety during the public health emergency. In: Proceedings of the 2024 3rd International Conference on Public Health and Data Science. New York, NY, USA: Association for Computing Machinery; 2025. pp. 169–74. 10.1145/3718677.3718704

[32] Gan WH, Lim JW, Koh D. Preventing Intra-hospital infection and transmission of coronavirus disease 2019 in Health-care workers. Saf Health Work. 2020;11:241–3. 10.1016/j.shaw.2020.03.001. 32292622 10.1016/j.shaw.2020.03.001 PMC7102575

[33] Zhu H, Yang X, Xie S, Zhou J. Prevalence of burnout and mental health problems among medical staff during the COVID-19 pandemic: a systematic review and meta-analysis. BMJ Open. 2023;13:e061945. 10.1136/bmjopen-2022-061945. 37474193 10.1136/bmjopen-2022-061945 PMC10360428

[34] Hemsley B, Balandin S, Worrall L. Nursing the patient with complex communication needs: time as a barrier and a facilitator to successful communication in hospital. J Adv Nurs. 2012;68:116–26. 10.1111/j.1365-2648.2011.05722.x. 21831131 10.1111/j.1365-2648.2011.05722.x

[35] Ganhao I, Trigo M, Paixao A. The impact of protective face masks and coverings on patient-health provider communication. Eur Psychiatry. 2021;64:S312–3. 10.1192/j.eurpsy.2021.840.

[36] Ehrlich H, McKenney M, Elkbuli A. Protecting our healthcare workers during the COVID-19 pandemic. Am J Emerg Med. 2020;38:1527–8. 10.1016/j.ajem.2020.04.024. 32336585 10.1016/j.ajem.2020.04.024 PMC7162741

[37] Gurita AV, Shehab AHIA, Luca L, Isabela N, Terpan M, Ciubara A. The consequences of the pandemic among patients with psychiatric history. Eur Psychiatry. 2022;65:S543–543. 10.1192/j.eurpsy.2022.1389.

[38] Morgner C. Trust and society: suggestions for further development of Niklas luhmann’s theory of trust. Can Rev Sociol. 2018;55:232–56. 10.1111/cars.12191. 29635830 10.1111/cars.12191

[39] Kupfer JM, Bond EU. Patient satisfaction and patient-centered care: necessary but not equal. JAMA. 2012;308:139–40. 10.1001/jama.2012.7381. 22782413 10.1001/jama.2012.7381

[40] De Benedictis A, Gualandi R, Saccoccia S, Pensieri C, Piredda M, De Micco F, et al. Back to the roots of nursing: qualitative study on the experience of nurses in the front line during the COVID-19 pandemic. Front Med (Lausanne). 2022;9:903517. 10.3389/fmed.2022.903517. 35755029 10.3389/fmed.2022.903517 PMC9231184

[41] Li J, Wang J, Kong X, Gao T, Wu B, Liu J, et al. Person-Centered communication between health care professionals and COVID-19-Infected older adults in acute care settings: findings from Wuhan, China. J Gerontol B Psychol Sci Soc Sci. 2021;76:e225–9. 10.1093/geronb/gbaa190. 33136158 10.1093/geronb/gbaa190 PMC7665773

[42] Oosthuizen I, Saunders GH, Manchaiah V, Swanepoel DW. Impact of SARS-CoV-2 virus (COVID-19) preventative measures on communication: A scoping review. Front Public Health. 2022;10:815259. 10.3389/fpubh.2022.815259. 35419343 10.3389/fpubh.2022.815259 PMC8995421

[43] Bu X, Wang Y, Du Y, Mu C, Zhang W, Wang P. Bridge the gap caused by public health crises: medical humanization and communication skills build a psychological bond that satisfies patients. Int J Equity Health. 2024;23:40. 10.1186/s12939-024-02116-4. 38409009 10.1186/s12939-024-02116-4 PMC10898071

[44] Skirbekk H, Magelssen M, Conradsen S. Trust in healthcare before and during the COVID-19 pandemic. BMC Public Health. 2023;23:863. 10.1186/s12889-023-15716-6. 37170208 10.1186/s12889-023-15716-6 PMC10173918

[45] Gopichandran V, Sakthivel K. Doctor-patient communication and trust in Doctors during COVID 19 times-A cross sectional study in Chennai, India. PLoS ONE. 2021;16:e0253497. 10.1371/journal.pone.0253497. 34161383 10.1371/journal.pone.0253497 PMC8221523

