# Correction: Divergent dynamics of nurse-patient trust during COVID-19 in China: the mediating effects of medical expectations and communication skills

**DOI:** 10.1186/s12912-025-04181-8

**Published:** 2025-12-01

**Authors:** Jingze Wang, Yaxuan Bi, Yanjiao Wang, Wenjing Yan, Yahui Yu, Pei Wang

**Affiliations:** 1 Faculty of Education, University of Maca, Macau, China; 2 Zhejiang Provincial Clinical Research Center for Mental Health, The Affiliated Kangning hospital of Wenzhou Medical University, Wenzhou, Zhejiang Province 325035 China; 3 School of Psychology, Inner Mongolia Normal University, Inner Mongolia Normal University, China; 4 Department of Pediatric Respiratory Medicine, The Second Affiliated Hospital of Wenzhou Medical University, Wenzhou, 325035 China; 5 College of Medical Humanities and Management, Wenzhou Medical University, North Central Road, Chashan Higher Education Park, Ouhai District, Wenzhou, Zhejiang Province 325035 China; 6 Key Research Center of Philosophy and Social Sciences of Zhejiang Province, Institute of Medical Humanities, Wenzhou Medical University, Wenzhou, 325035 China; 7 College of Education, College of Education, Shanghai, 210034 China; 8 School of Mental Health, Wenzhou Medical University, Wenzhou, 325000 China

**Correction to:** ***BMC Nurs*** ***24***, ***1359 (2025).***

10.1186/s12912-025-03988-9.

In this article, Wenjing Yan should have been denoted as a corresponding author.

The original article [1] has been corrected.
